# Signal-To-Noise Crossover Dose: Sand et al. Respond

**DOI:** 10.1289/ehp.1205212R

**Published:** 2012-07-02

**Authors:** Salomon Sand, Christopher J. Portier, Daniel Krewski

**Affiliations:** Risk Benefit Assessment Department, National Food Administration, Uppsala, Sweden, E-mail: salomon.sand@slv.se; National Center for Environmental Health, Agency for Toxic Substances and Disease Registry, Atlanta, Georgia; McLaughlin Centre for Population Health Risk Assessment, University of Ottawa, Ottawa, Ontario, Canada

We appreciate the opportunities highlighted by Chiu et al. to augment the concept of the signal-to-noise crossover dose (SNCD) (Sand et al. 2011) with biological consideration. In similarity to our interpretation of the SNCD concept, Chiu et al. note that our approach provides a transparent and objective method to demark where extrapolation begins and that it appears consistent with the intent of the point of departure (POD) to characterize “the beginning of extrapolation to lower doses” (U.S. Environmental Protection Agency 2005).

In our article (Sand et al. 2011), we compared the SNCD with traditional PODs used in risk assessment [i.e., the benchmark dose (BMD) and the no observed adverse effect level (NOAEL)]. In addition, we compared human exposure guidelines [generally referred to as reference doses (RfDs) in our article] resulting from using the SNCD, the BMD, and the NOAEL as PODs. The SNCD-based exposure guideline was derived by linear extrapolation from the upper bound on extra risk at the SNCD (UER_SNCD_) down to a target risk of 1/1,000. When considering new risk assessment strategies, an initial step is to address how they compare against more traditional approaches. The specific settings we used in the derivation of the SNCD-based exposure guideline were selected primarily as a result of such considerations; that is, using these settings, the SNCD concept can be compared to more standard BMD and NOAEL approaches at the level of the human exposure guideline in a calibrated manner, providing a starting point for further discussion.

In our article (Sand et al. 2011) we suggested that further development of the SNCD concept could involve the use of alterna-tive target risks (e.g., based on public health con-siderations, as well as alternative low-dose extrapolation models), and we also pointed out that animal-to-human extrapolation may be regarded as a separate step after the SNCD-based POD has been established. This appears to be in line with the developments proposed by Chiu et al. in their letter. Generally speaking, they suggest an approach that separately takes into account biological considerations related to the severity of the end point via the target effect level (TEL), statistical considerations related to the study data via the UER_SNCD_, and adjustments from the test species to sensitive humans, after the POD associated with the TEL has been derived, via uncertainty factors or chemical-specific adjustments.

Chiu et al. suggests in more detail that if UER_SNCD_ < TEL, the POD corresponding to that TEL can be derived directly, whereas the case UER_SNCD_ > TEL indicates that low-dose extrapolation from the SNCD is required to reach the dose associated with the TEL (where UER_SNCD_/TEL measures the extent of extrapolation). In principal, this appears reasonable provided that the SNCD concept is generalized to any type of response data (i.e., not only cancer, but continuous or quantal end points in general). The SNCD may then represent a starting point for low-dose extrapolation when the upper bound on risk (extra risk) or continuous effect (e.g., relative effect) at the SNCD is higher than what is considered acceptable from a biological or risk management point of view; although TELs should ideally be fully biologically based, a certain level of policy is likely to be involved, including use of default values. It remains to be seen in practice how the SNCD compares with PODs corresponding to default benchmark response (BMR) levels, TELs, or other such measures, for various types of response data.

We are in the process of extending the comparison of different PODs performed in our previous study (Sand et al. 2011) to the case of high -throughput screening data. Accounting for the severity of effect for such data is a major challenge, for example, using a TEL/BMR concept or perhaps requiring development of multivariate/multidimensional extensions thereof. We agree that separating biological and statistical considerations will enhance the transparency and consistency of chemical assessments.
